# Decision-making factors affecting different family members regarding the placement of relatives in long-term care facilities

**DOI:** 10.1186/1472-6963-14-21

**Published:** 2014-01-17

**Authors:** Ying-Chia Huang, Chiao-Lee Chu, Ching-Sung Ho, Shou-Jen Lan, Chen-Hsi Hsieh, Yen-Ping Hsieh

**Affiliations:** 1 Department of Healthcare Administration, Asia University, Taichung City, Taiwan; 2 Department of Long Term Care, National Quemoy University, Quemoy City, Taiwan; 3 Department of Medicine, School of Medicine, National Yang-Ming University, Taipei City, Taiwan; 4 Department of Radiology, Division of Radiation Oncology, Far Eastern Memorial Hospital, Taipei City, Taiwan; 5 Department of Senior Citizen Service Management, National Taichung University of Science and Technology, No. 193, Sec. 1, Sanmin Rd., Taichung City, Taiwan

**Keywords:** Decision making, Long-term care facilities, Placement, Spouses, Children, Grandchildren, Relatives

## Abstract

**Background:**

The aim of this research was to investigate factors affecting different family members’ decisions regarding the placement of relatives in long-term car (LTC) facilities in Taiwan. The objective was to investigate the correlations between family members’ personal traits, the living conditions of residents in the LTC facilities, and family members’ experiences with LTC facilities.

**Methods:**

This study selected family members visiting residents in LTC facilities as research subjects and used a structured questionnaire to perform face-to-face interviews. This study used nonlinear canonical correlation analysis (OVERALS) to categorize the decision-making factors affecting family members’ choices of LTC facilities.

**Results:**

The results showed that when making decisions about the placement of family members, spouses chose facilities according to their own life experiences, children considered medical treatment convenience, grandchildren preferred to collect relevant information on facilities, and other relatives preferred to decide based on introductions from government departments.

**Conclusions:**

These results help clarify how different family roles affect decision-making processes regarding the choice of LTC facilities. In particular, spouses and female relatives require an interventional service mechanism that provides consultation or referral information.

## Background

This study investigated differences in the choice of long-term care (LTC) facilities based on different family roles to expand the understanding of relatives’ perspectives on such facilities. Different family members have different types of burdens in caring for family members. Spouses bear greater care and financial burdens [1-3], while adult children provide emotional support and unpaid work [1,4]. In addition, caregiving responsibilities borne by children vary based on gender [5,6]. Elderly who do not have children, or whose children have died, receive care and emotional support through informal resources such as neighbors, living kin, and nongovernmental organizations [7,8].

Many studies have shown that the main reason family members place relatives in LTC facilities is because they cannot provide the necessary care [9-11]. During the placement process, however, family caregivers experience pressure and negative emotions [12-15]. Few studies have examined how family members choose LTC facilities. Such studies have found that children are likely to visit several LTC facilities and decide based on word of mouth. In addition, children attach importance to factors such as distance from home, cost, caregiver-to-patient ratio, environmental cleanliness, and service quality [16,17].

Most studies have used qualitative research methods to investigate the decision-making burdens and conflicts faced by family members and the elderly regarding placement and the placement process. Few studies, however, have investigated how children choose and assess LTC facilities, and even fewer have investigated how different family roles, personal traits, and personal experiences with LTC facilities might affect the choice of an LTC facility. Such a perspective is especially important in Asia since the placement decision is usually made by family members after discussing their thoughts. When family members are in agreement, they will aggressively provide support during the placement process. If there is disagreement, however, it is more difficult to make a decision regarding placement [14,18,19]. An understanding of the factors that affect different family members in choosing an LTC facility can help facilitate the provision of relevant care resources and interventional service mechanisms during the decision-making process.

The subjects of this study were the family members of residents in LTC facilities. The aim of this research was to investigate factors affecting different family members’ decisions regarding the placement of relatives in LTC facilities in Taiwan. This study used nonlinear canonical correlation analysis (OVERALS) to categorize the decision-making factors affecting family members’ choices of LTC facilities.

## Methods

This study selected the relatives of residents in LTC facilities as the research subjects. LTC facilities in Taiwan can be divided into two main types: senior citizen welfare institutions (SCWIs), which are supervised by the Department of Social Affairs, and nursing homes (NHs), which are supervised by the Department of Health. Both types of facilities provide daily care and general nursing care. Residents of NHs have a more severe degree of disability than those living in SCWIs. This study distributed questionnaires in 180 LTC facilities (111 SCWIs and 69 NHs) between January and April 2010 and performed face-to-face interviews with subjects for approximately 20–30 minutes. It was difficult to personally obtain information on LTC residents’ family members; therefore, we asked the facilities to provide access to the family members of residents for a period of more than three months. The subjects included in the study were the residents’ spouses, children, grandchildren, or other relatives. The researcher contacted 402 family members of residents, as provided by the LTC facilities, and explained the purpose of the study. A total of 286 family members agreed to participate. Eight of them could not complete the interviews for personal reasons, resulting in a total of 278 valid questionnaires.

This study was approved by the Medical Ethics Committee of Asia University (No. 1006008). The questionnaire contents were sent to the LTC facilities that agreed to participate for review and approval. The family members received written information regarding the research objectives. Letters of consent were obtained from those who agreed to participate. All interviewers were trained prior to conducting the surveys.

### Measures

In accordance with the results of existing studies, this study found that the personal traits of family members, the living conditions of residents in the LTC facilities, and family members’ experiences with LTC facilities were associated with the decision-making factors for placing relatives in LTC facilities [1,3,14,17-21]. The questionnaire (see Additional file 1) [1,3,14,17-22] was divided into three sets, with the variables and category symbols shown in Additional file 2: Tables S1.1-S1.3.

Set A consisted of the family members’ demographic characteristics, including gender (V1), age (V2), education level (V3), marital status (V4), self-perceived financial status (V5), and relationship to LTC resident (V6).

Set B consisted of questions regarding the current living conditions of residents, including the LTC facility type (V7), the number of beds in the facility (V8), and the length of time the resident had lived in the facility (V9). Items V10–V14 were intended to determine who paid for the LTC facilities: “paid by resident” (V10), “paid by spouse” (V11), “paid by children” (V12), “paid by relative” (V13), and “paid by the government” (V14). V15 asked questions regarding how family members learned of the LTC facilities and where the residents currently lived: “propaganda from the LTC facilities”, “introduction from friends and relatives”, “introduction from hospital-related personnel”, “LTC facility’s proximity to home”, “introduction from governmental units”, and “others”. Items V16–V19 sought to confirm the top four reasons family members placed residents in the LTC facilities where they currently lived: “LTC facility’s proximity to home” (V16), “convenience for family members to visit the resident” (V17), “service quality of the facility” (V18), and “medical treatment convenience” (V19).

Set C consisted of family members’ experiences with LTC facilities, including “reason for contact with the LTC facility” (V20) and “experiences visiting LTC facility” (V21). Items V22–V24 were intended to determine the types of LTC facilities family members had visited: “type of LTC facility visited: nursing home” (V22), “type of LTC facility visited: senior citizen welfare institution” (V23), and “type of LTC facility visited: community care facility” (V24). Items V25–V28 were intended to confirm the four service-quality items to which the family members attached the most importance: “environmental cleanliness” (V25), “lighting of the rooms in the LTC facility” (V26), “ventilation of the LTC facility” (V27), and “safety of the LTC facility” (V28).

### Analysis

This study used SPSS for Windows 12.0 to perform the analyses. The mean, standard deviation, percentage, and nonlinear canonical correlation analysis (OVERALS) were used in the calculations. OVERALS is a technique for canonical correlation analysis using two or more sets of variables. The data measurement levels that can be processed by OVERALS include numerical, ordinal, and nominal levels, which can be separately defined for each variable. The OVERALS technique searches for commonalities between different sets of variable measurements of the same objects [23]. The purpose is to determine how similar the sets of variables are to each other.

OVERALS can analyze the loss index, eigenvalues, fit index, weight index, multiple fit indices, and the component loading index as established for each variable and then draw a two-dimensional graph for each variable. OVERALS uses the alternating least squares (ALS) algorithm, which makes it possible to calculate fit function and loss function. A perfect adaptation would comply with the number of chosen dimensions, where the maximum number of dimensions matches the sum of all linear combinations of variable characteristics from the sets. The loss function states the difference between the numbers of chosen dimensions that best calculates adaptation. Moreover, the eigenvalues are calculated, which can be determined by analyzing data from the fit and loss functions. These eigenvalues indicate to what extent each dimension accounts for the loss function, as compared to the calculated correlation, and can assume values between 0 and 1. The group of variables in a set should be based on theoretical considerations since the combination of variables is of higher importance in OVERALS than each variable itself. Single variables are only important when they contain information independent of the information of other variables in the same set [23,24].

In total, 28 variables with either nominal or ordinal scaling levels were included in the analysis (Additional file 2: Tables S1.1-S1.3). The interpretation of these coherences should be based on the chosen dimensions, which represent a common level of analysis for all variables running along the respective dimension. OVERALS was used to construct the similarities between the three sets [23,24].

## Results

Additional file 2: Table S1.1 shows the data for the demographic characteristics of the family members (Set A). Educational attainment was mainly college level (40.29%), self-perceived financial status was mainly “ordinary” (78.06%), and the age range was mainly 51–60 (30.58%). Roughly 60% of the family members were the children of the residents (66.55%), followed by spouses (11.51%) and other relatives (14.39%).

Additional file 2: Table S1.2 shows the data regarding the living conditions of the residents (Set B). More residents lived in SCWIs (52.16%) than in NHs (47.84%). Most lived in small LTC facilities with fewer than 49 beds (58.99%), and most had lived in the LTC facilities for 1–2 years (41.37%). Payment for the LTC facilities was mostly made by the children (75.54%), followed by the government (10.79%). The family members learned of the LTC facilities through introductions from relatives and friends (56.83%) and hospital-related personnel (23.02%). The main reasons for choosing the LTC facility were as follows: proximity to home (66.19%), convenience for visiting the resident (65.47%), and the service quality of the facility (39.93%).

Additional file 2: Table S1.3 concerns family members’ experience with LTC facilities (Set C). Most had visited LTC facilities to “choose an LTC facility for their family member” (90.29%). However, 47.48% of family members had never visited an LTC facility. The family members who had visited LTC facilities mainly visited SCWIs (41.01%). Family members attached the most importance to environmental cleanliness (56.83%) and the lighting of the rooms (18.71%) when choosing an LTC facility.

This study used OVERALS to investigate the correlations among the three sets.

A two-dimensional solution was chosen to visually map the constructed space using OVERALS with the described three sets and their variables. The two dimensions produced a fit of 1.172. The fit shows the extent to which the OVERALS solution fits the optimally quantified data with regard to the association between the sets.

This two-dimensional solution appears justified since the eigenvalues of the first two dimensions were quite high: the first dimension had an eigenvalue of 0.603, the highest explanatory power showing the coherence of sets among each other; the second dimension had an eigenvalue of 0.569. The eigenvalues of the two dimensions add up to a fit of 1.172; thus, they can be interpreted as a proportion of the explained variance.

**Figure 1:**
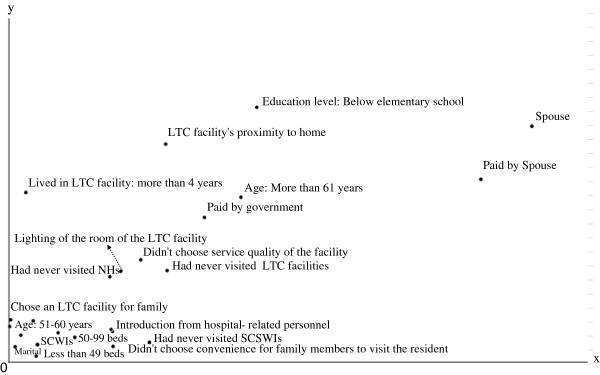
Centroid plots: Quadrant I.

**Figure 2:**
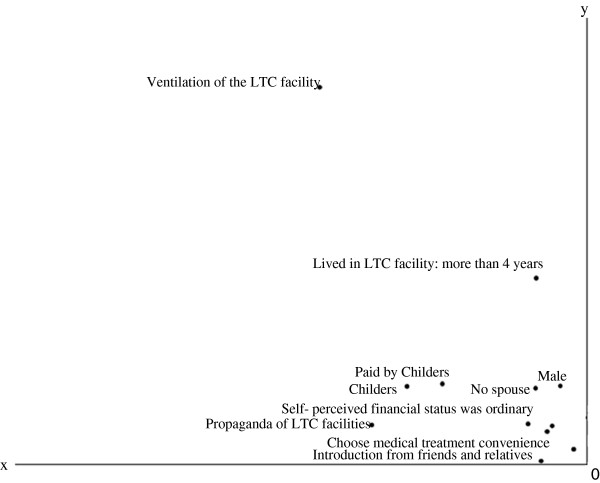
Centroid plots: Quadrant II.

**Figure 3:**
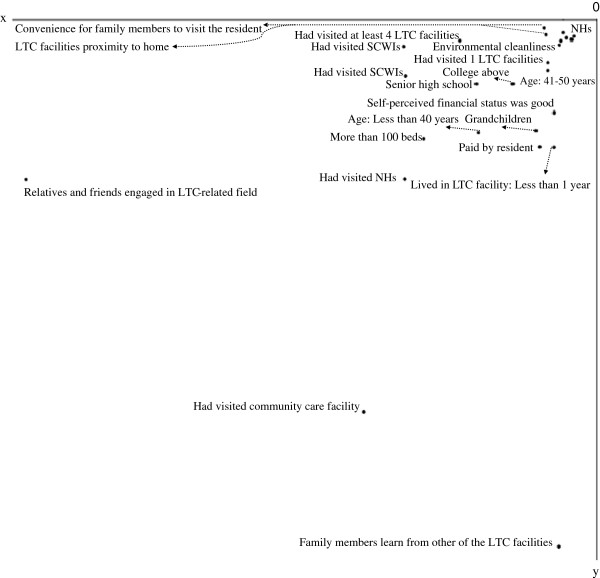
Centroid plots: Quadrant III.

**Figure 4:**
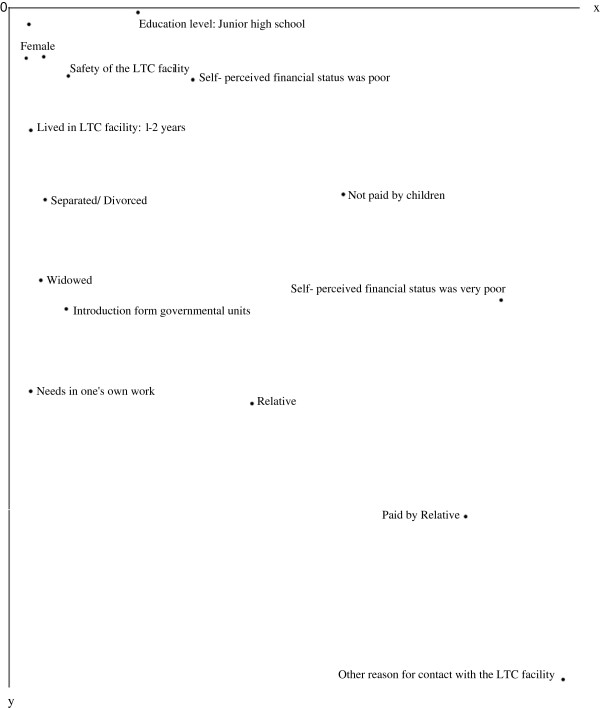
Centroid plots: Quadrant IV.

Figures 1, 2, 3 and 4 show the centroid plots by the variables. The positions of the projected centroids determined the interpretation of the direction of the variables in the four quadrants.

Figure 1 shows that the family members in Quadrant I were mainly spouses (v6.1) aged 51 and above (v2.3 and v2.4) with an educational level of elementary school (v3.1). Their family members lived in SCWIs (v7.2) with fewer than 99 beds (v8.1 and v8.2) for more than 4 years (v9.4). Payments for the LTC facilities were made by spouses (v11.2) and the government (v14.2). They learned of the LTC facilities through introduction by hospital-related personnel (v15.3) and proximity to home (v15.4). They contacted LTC facilities because they intended to help their families choose LTC facilities (v20.1); however, they had never visited LTC facilities (v21.1). The family members in this quadrant attached importance to the lighting of the rooms when choosing an LTC facility (v26.2).

Figure 2 shows that the family members in Quadrant II were mainly the unmarried sons of residents (v1.1 and v4.1); their self-perceived financial status was “ordinary” (v5.2). Their family members had lived in LTC facilities for less than three years (v9.3). Payments for the LTC facilities were made by the children (v12.2). They learned of the LTC facilities through relatives, friends, and propaganda from the LTC facilities (v15.1 and v15.2). They chose LTC facilities based on the convenience of medical treatment (v19.2). They attached importance to ventilation when choosing an LTC facility (v27.2).

Figure 3 shows that The family members in Quadrant III were mainly grandchildren aged 50 and under (v2.1, v2.2, and v6.3), with educational levels of high school, college, and above (v3.3 and v3.4); their self-perceived financial status was “good” (v5.1). Their families had lived in NHs (v7.1) with at least 100 beds (v8.3) for less than 1 year (v9.1). Payments for the facilities were made by the residents themselves (v10.2). The family members chose the LTC facilities based on their proximity to home, visiting convenience, and quality (v16.2, v17.2, and v18.2). Their opportunities for contacting LTC facilities came through relatives and friends engaged in LTC-related fields (v20.3). These family members had visited at least one LTC facility, including NHs, SCWIs, and community care facilities (v21.2, v21.3, v21.4, v22.2, v22.3, and v24.2). They attached importance to environmental cleanliness when choosing an LTC facility (v25.2).

Figure 4 shows that The family members in Quadrant IV were mainly grandchildren aged 50 and under (v2.1, v2.2, and v6.3), the family members were female relatives (v1.2 and v6.4). Their educational level was junior high school (v3.2), their marital status was widowed or divorced (v4.3 and v4.4), and their self-perceived financial status was poor (v5.3 and v5.4). Their families had lived in LTC facilities for less than two years (v9.2). Payments were made by relatives (v13.2). The family members learned of the LTC facilities through introduction by government units (v15.5). They contacted the LTC facilities because of their work needs and other reasons (v20.2). They attached importance to safety when choosing an LTC facility (v28.2).

## Discussion

This study investigated the influence of differences in family member roles on the decision-making process in choosing an LTC facility. Previous studies mainly used qualitative analyses to investigate children’s decision-making processes regarding the choice of an LTC facility [14,16,17]. However, in addition to discussing the influence of differences in family member roles, this study used an OVERALS analysis to categorize the decision-making factors that affect a family member’s choice of LTC facility.

In Quadrant I, the results of the personal data for spouses are consistent with those of previous studies: older age, lower education level, and payment by either the spouse or the government [3]. Studies of spousal care load have indicated that spouses less frequently seek assistance from others and use care resources [1,3]. These characteristics of spousal care are also reflected in our results: spouses preferred familiar regions (they chose LTC facilities near their homes or through introduction by hospital-related personnel), and they chose according to their life experiences. The distance between an LTC facility and the spouse’s home might be an important factor to consider.

The spouses had never visited LTC facilities. When faced with placement, they tended to make hasty decisions, which could be one of the reasons for their lack of information on LTC facilities [14,25]. The results showed that when faced with making placement decisions, spouses required the intervention of relevant consultation services.

The family members in Quadrant II were mainly the residents’ children; they were single sons paid for the LTC facilities. This result aligns with other relevant studies on the placement process in Asia. Filial piety in Chinese culture results in children providing financial support for their parents [26-28]. In addition to considering LTC facilities through word of mouth from relatives and friends [16,17], children also referred to information provided by the LTC facilities. The main difference between our findings and those of previous studies concerns the reason for choosing an LTC facility—namely, “medical treatment convenience” as opposed to factors such as caregiver-to-patient ratio and environmental cleanliness [26-28]. In Chinese society, when the physical functioning of elderly people deteriorates, their children move them from home care to an LTC facility (Hsu, 2012). Consequently, the factor affecting the children’s final choice of an LTC facility is the distance between the LTC facility and the hospital for medical treatment convenience.

The family members in Quadrant III were mainly grandchildren aged 50 and under whose educational level and self-perceived financial status were “good”. The reasons for placement in LTC facilities were similar to the findings of previous studies, including proximity to home, convenience for family members to visit residents, and service quality [16,17,25]. The results showed that when the LTC facilities were paid for by the residents themselves, family members mainly attached importance to service quality and the distance from home. Moreover, the grandchildren in this quadrant collected information related to LTC facilities. In addition to receiving information from relatives and friends engaged in LTC-related fields, grandchildren also visited many different types of LTC facilities. There is a lack of research investigating the role of grandchildren in making decisions about placement. This study speculated that family members in Asia mainly discuss comments made when making a decision about placement [14,18,19]. As a result, grandchildren who are younger tend to play the role of information collectors.

The family members in Quadrant IV were mainly female relatives engaged in LTC-related fields. Their educational level was low, they were poor, and they were divorced or widowed, suggesting a lack of financial support and resources from relatives or families. However, the people paying for the LTC facilities for this quadrant were other relatives. Relevant studies investigating elderly people who do not have children have indicated that distant relatives often provide care [7,8]. Relatives in this quadrant arranged for placement in LTC facilities based on introduction by the government. If the relatives themselves did not have sufficient resources, they sought information on LTC facilities provided by the government.

Different family members attach importance to different aspects of LTC facilities. Spouses attach importance to the lighting of rooms, children attach importance to ventilation, grandchildren attach importance to overall environmental cleanliness, and other relatives attach importance to safety. It seems, however, that the items to which family members attach importance do not affect the decision-making process for choosing an LTC facility. The results showed that family members tried their best to make placement decisions from the perspective of their family and according to their own resources. This result is similar to the findings of existing studies [2,14,28].

### Limitations

Certain aspects of this study could be improved in future studies. First, the data were collected from family members on lists offered by the LTC facilities. The listed family members might have been those who interacted well with LTC facilities; this might have created bias in the sample selection and limited the generalizability of the results. Future studies should conduct surveys on the current status of family members of residents in LTC facilities. Second, this study investigated the personal traits of family members, the living conditions of residents in the LTC facilities, and family members’ experiences with LTC facilities. The results clearly showed that differences in family member roles led to differences in the decision-making process over the choice of LTC facility; this helps clarify that different service interventions can be provided during the placement of relatives. However, the decision-making process for the placement of relatives is affected by many other factors, including the amount of money actually paid to LTC facilities, birth order, gender of the family members, past caregiving experiences with family members, and the implementation status of caregiving policy or medical policy. Future studies should investigate the placement of relatives in LTC facilities from multiple perspectives.

## Conclusions

This study found that different family members chose LTC facilities for different reasons. Spouses decided according to their own life experiences, children took into account medical treatment convenience, grandchildren preferred collecting information on LTC facilities, and female relatives decided according to introductions from the government. Because of their lack of care habits or information, spouses and female relatives in particular require an interventional service mechanism that provides consultation or referral information.

## Competing interests

We have no personal or financial conflicts of interest to declare. We have not entered into any agreement that could interfere with our access to the research data or our ability to analyze the data, prepare manuscripts, and publish them.

## Authors’ contributions

YPH conceived of the study, analyzed the data, and drafted the initial manuscript. YCH conceived of the study, analyzed the data, and critically reviewed the manuscript. CLC and CSH analyzed the data and critically reviewed the manuscript. SJL and CHH reviewed and edited the manuscript. All authors read and approved the final manuscript.

## Pre-publication history

The pre-publication history for this paper can be accessed here:

http://www.biomedcentral.com/1472-6963/14/21/prepub

## Supplementary Material

Additional file 1 **Questionnaire.** Long-Term Care Resident’s Family Member’s Opinions on Choosing LTC Facilities. Click here for file

Additional file 2: Table S1.1 Basic information of Sets of variables, variables, scale type and category symbols: Set A. **Table S1.2.** Basic information of Sets of variables, variables, scale type and category symbols: Set B. **Table S1.3.** Basic information of Sets of variables, variables, scale type and category symbols: Set C. Click here for file
