# Supplementary material for: Decision-making factors affecting different family members regarding the placement of relatives in long-term care facilities
Source: BMC Health Serv Res. 2014 Jan 17;14:21. doi: 10.1186/1472-6963-14-21 (PMC3897917; doi:10.1186/1472-6963-14-21)
Supplement: Additional file 1 — Questionnaire. Long-Term Care Resident’s Family Member’s Opinions on Choosing LTC Facilities. [file 1472-6963-14-21-S1.doc]

Additional file 1: Appendix

**Long-Term Care Resident’s Family Member’s Opinions on Choosing LTC Facilities**

**(Ⅰ) Family member's demographic characteristics**

1. Gender: □1.Male □2.Female

2. Age:

□1.Less than 40 years □2.41-50 years

□3.51-60 years □4.More than 61 years

3. What is your educational level?

□1.Below elementary school □2.Junior high school

□3.Senior high school □4.College and above

4. What is your marital status?

□1. No spouse □2. Married

□3. Widowed □4. Separated / divorced

5. What is your self-perceived financial status?

□1. Good □2. Ordinary

□3. Poor □4. Very poor

6. What is your relationship with the resident in the LTC facility?

□1. Spouse □2. Children

□3. Grandchildren □4. Relative

**(Ⅱ) Current Status of Resident Living in LTC facility**

7. What is the LTC facility type that your family member lives in?

□1. Nursing Home □2. Senior Citizen Welfare Institution

8. How many beds are there in the LTC facility where your family member lives?

□1.Fewer than 49 beds □2. 50-99 beds □3. More than 100 beds

9. How long has your family member lived in the LTC facility?

□1. Less than 1 year □2. 1-2 years

□3. 2-3 years □4. More than 4 years

10. Who pays for the expense of the LTC facility?

10.1 Paid by the resident □1. No □2. Yes

10.2 Paid by the spouse □1. No □2. Yes

10.3 Paid by the children □1. No □2. Yes

10.4 Paid by a relative □1. No □2. Yes

10.5 Paid by the government □1. No □2. Yes

11. How did family members learn about the LTC facility?

□1. Advertising of the LTC facility □2. Referred by friends and relatives

□3. Referred by hospital-related personnel □4. LTC facility is close to home

□5. Referred by governmental units □6. Other

12. Was the LTC facility chosen because of its proximity to home?

□1. No □2. Yes

13. Was the LTC facility chosen because of the convenience for family members to visit the resident?

□1. No □2. Yes

14. Was the LTCA facility chosen because of its service quality?

□1. No □2. Yes

15. Was the LTC facility chosen because of its medical treatment convenience?

□1. No □2. Yes

**(Ⅲ) Family member’s experiences in contact with the LTC facilities**

16. Why did you contact the LTC facility?

□1. Chose an LTC facility for family □2. Needed in one’s own work

□3. Relatives and friends engaged in LTC-related field □4. Other

17. What’s your experience in visiting the LTC facilities?

□1. Had never visited LTC facilities □2. Had visited 1 LTC facilities

□3. Had visited fewer than 3 LTC facilities □4. Had visited at least 4 LTC facilities

18. What type of LTC facilities did you visit?

18.1 Visited nursing homes □1. No □2. Yes

18.2 Visited Senior Citizen Welfare Institutions □1. No □2. Yes

18.3 Visited community care facility □1. No □2. Yes

19. Do you pay much attention to the cleanliness of LTC facilities?

□1. No □2. Yes

20. Do you pay much attention to the lighting in the rooms of the LTC facilities?

□1. No □2. Yes

21. Do you pay much attention to the ventilation of the LTC facilities?

□1. No □2. Yes

22. Do you pay much attention to the safety of the LTC facilities?

□1. No □2. Yes
