# Supplementary material for: Decision-making factors affecting different family members regarding the placement of relatives in long-term care facilities
Source: BMC Health Serv Res. 2014 Jan 17;14:21. doi: 10.1186/1472-6963-14-21 (PMC3897917; doi:10.1186/1472-6963-14-21)
Supplement: Additional file 2: Table S1.1 — Basic information of Sets of variables, variables, scale type and category symbols: Set A. Table S1.2. Basic information of Sets of variables, variables, scale type and category symbols: Set B. Table S1.3. Basic information of Sets of variables, variables, scale type and category symbols: Set C. [file 1472-6963-14-21-S2.doc]

| Table 1.1 Basic information of Sets of variables, variables, scale type and category symbols: Set A | | | | |
| --- | --- | --- | --- | --- |
| Set of variables / Category symbols | | Scale type | N | Percentage |
| ***Set A: Family members’ demographic characteristics*** | | | | |
| **V1 Gender** | |  |  |  |
| v1.1 | Male | Nominal | 128 | 46.04 |
| v1.2 | Female |  | 150 | 53.96 |
| **V2 Age** | |  |  |  |
| v2.1 | less than 40 years | Ordinal | 72 | 25.90 |
| v2.2 | 41-50 years |  | 68 | 24.46 |
| v2.3 | 51-60 years |  | 85 | 30.58 |
| v2.4 | More than 61 years |  | 53 | 19.06 |
| **V3 Education level** | |  |  |  |
| v3.1 | Below elementary school | Nominal | 47 | 16.91 |
| v3.2 | Junior high school |  | 35 | 12.59 |
| v3.3 | Senior high school |  | 84 | 30.22 |
| v3.4 | College above |  | 112 | 40.29 |
| **V4 Marital status** | |  |  |  |
| v4.1 | No spouse | Nominal | 54 | 19.42 |
| v4.2 | Marital status |  | 198 | 71.22 |
| v4.3 | Widowed |  | 14 | 5.04 |
| v4.4 | Separated / divorced |  | 12 | 4.32 |
| **V5 Self-perceived financial status** | | | | |
| v5.1 | Good | Nominal | 18 | 6.47 |
| v5.2 | Ordinary |  | 217 | 78.06 |
| v5.3 | Poor |  | 35 | 12.59 |
| v5.4 | Very poor |  | 8 | 2.88 |
| **V6 relationship with residents in LTC facilities** | | | | |
| v6.1 | Spouse | Ordinal | 32 | 11.51 |
| v6.2 | Childres |  | 185 | 66.55 |
| v6.3 | Grandchildren |  | 21 | 7.55 |
| v6.4 | Relative |  | 40 | 14.39 |

| Table 1.2 Basic information of Sets of variables, variables, scale type and category symbols: Set B | | | | |
| --- | --- | --- | --- | --- |
| Set of variables / Category symbols | | Scale type | N | Percentage |
| ***Set B: Current living conditions of residents*** | |  |  |  |
| **V7 LTC facility type** | |  |  |  |
| v7.1 | NHs | Nominal | 133 | 47.84 |
| v7.2 | SCWIs |  | 145 | 52.16 |
| **V8 The number of beds in the LTC facility** | | | |  |
| v8.1 | Less than 49 beds | Ordinal | 164 | 58.99 |
| v8.2 | 50-99 beds |  | 69 | 24.82 |
| v8.3 | More than 100 beds |  | 45 | 16.19 |
| **V9 The length of time the resident had lived in the LTC facility** | | | | |
| v8.1 | Less than 1 year | Ordinal | 39 | 14.03 |
| v8.2 | 1-2 year |  | 115 | 41.37 |
| v8.3 | 2-3 years |  | 52 | 18.71 |
| v8.4 | More than 4 years |  | 72 | 25.90 |
| **V10 Paid by resident** | |  |  |  |
| v10.1 | No | Nominal | 232 | 83.45 |
| v10.2 | Yes |  | 46 | 16.55 |
| **V11 Paid by spouse** | |  |  |  |
| v11.1 | No | Nominal | 259 | 93.17 |
| v11.2 | Yes |  | 19 | 6.83 |
| **V12 Paid by children** | |  |  |  |
| v12.1 | No | Nominal | 68 | 24.46 |
| v12.2 | Yes |  | 210 | 75.54 |
| **V13 Paid by relative** | |  |  |  |
| v13.1 | No | Nominal | 263 | 94.60 |
| v13.2 | Yes |  | 15 | 5.40 |
| **V14 Paid by the government** | |  |  |  |
| v14.1 | No | Nominal | 248 | 89.21 |
| v14.2 | Yes |  | 30 | 10.79 |
| **V15 How family members learn of the LTC facilities** | | | |  |
| v15.1 | Propaganda of LTC facilities |  | 23 | 8.27 |
| v15.2 | Introduction from friends and relatives |  | 158 | 56.83 |
| v15.3 Introduction from hospital-related personnel Nominal | | | 64 | 23.02 |
| v15.4 | LTC facility’s proximity to home |  | 18 | 6.47 |
| v15.5 | Introduction form governmental units |  | 5 | 1.80 |
| v15.6 | Other |  | 10 | 3.60 |
| **V16 LTC facility’s proximity to home** | |  |  |  |
| v16.1 | No | Nominal | 94 | 33.81 |
| v16.2 | Yes |  | 184 | 66.19 |
| **V17 Convenience for family members to visit the resident** | | | | |
| v17.1 | No | Nominal | 96 | 34.53 |
| v17.2 | Yes |  | 182 | 65.47 |
| **V18 Service quality of the facility** | |  |  |  |
| v18.1 | No | Nominal | 111 | 39.93 |
| v18.2 | Yes |  | 167 | 60.07 |
| **V19 Medical treatment convenience** | |  |  |  |
| v19.1 | No | Nominal | 167 | 60.07 |
| v19.2 | Yes |  | 111 | 39.93 |

Abbreviations: NHs, nursing home; SCWIs, senior citizen welfare institutions

| Table 1.3 Basic information of Sets of variables, variables, scale type and category symbols: Set C | | | | |
| --- | --- | --- | --- | --- |
| Set of variables / Category symbols | | Scale type | N | Percentage |
| ***Set C: Family members’ experiences of contact with LTC facilities*** | | | | |
| **V20 Reason for contact with the LTC facility** | |  |  |  |
| v20.1 | Chose an LTC facility for family | Nominal | 251 | 90.29 |
| v20.2 | Needs in one’s own work |  | 16 | 5.76 |
| v20.3 Relatives and friends engaged in LTC-related field | | | 6 | 2.16 |
| v20.4 | Other |  | 5 | 1.80 |
| **V21 Experiences of visiting LTC facility** | |  |  |  |
| v21.1 | Had never visited LTC facilities | Ordinal | 132 | 47.48 |
| v21.2 | Had visited 1 LTC facilities |  | 30 | 10.79 |
| v21.3 | Had visited less than 3 LTC facilities |  | 83 | 29.86 |
| v21.4 | Had visited at least 4 LTC facilities |  | 33 | 11.87 |
| **V22 Type of the LTC facility visited: NHs** | |  |  |  |
| v22.1 | No | Nominal | 185 | 66.55 |
| v22.2 | Yes |  | 93 | 33.45 |
| **V23 Type of the LTC facility visited: SCWIs** | |  |  |  |
| v23.1 | No | Nominal | 164 | 58.99 |
| v23.2 | Yes |  | 114 | 41.01 |
| **V24 Type of the LTC facility visited: community care facility** | | | | |
| v24.1 | No | Nominal | 253 | 91.01 |
| v24.2 | Yes |  | 25 | 8.99 |
| **V25 Environmental cleanliness** | |  |  |  |
| v25.1 | No | Nominal | 120 | 43.17 |
| v25.2 | Yes |  | 158 | 56.83 |
| **V26 Lighting of the room of the LTC facility** | |  |  |  |
| v26.1 | No | Nominal | 226 | 81.29 |
| v26.2 | Yes |  | 52 | 18.71 |
| **V27 Ventilation of the LTC facility** | |  |  |  |
| v27.1 | No | Nominal | 237 | 85.25 |
| v27.2 | Yes |  | 41 | 14.75 |
| **V28 Safety of the LTC facility** | |  |  |  |
| v28.1 | No | Nominal | 238 | 85.61 |
| v28.2 | Yes |  | 40 | 14.39 |
